# Supplementary figures and images for: Predicting the Non-Deterministic Response of a Micro-Scale Mechanical Model Using Generative Adversarial Networks
Source: Materials (Basel). 2022 Jan 26;15(3):965. doi: 10.3390/ma15030965 (PMC8838419; doi:10.3390/ma15030965)

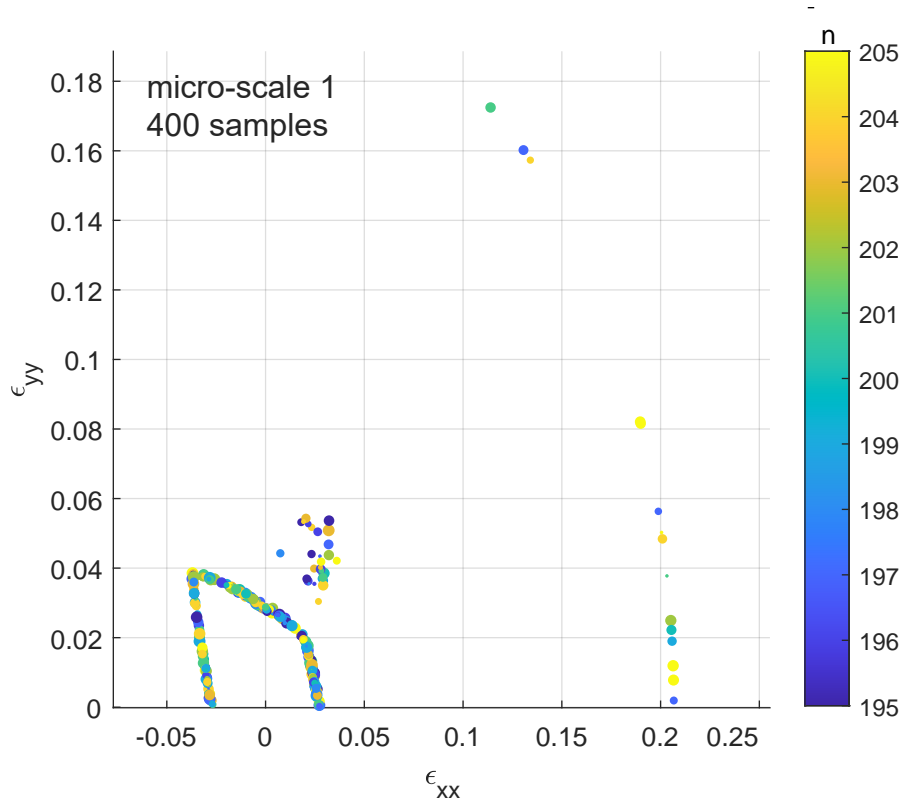

Supplement: Supplementary file 1 [file materials-15-00965-s001.zip › Figure S1a.pdf]

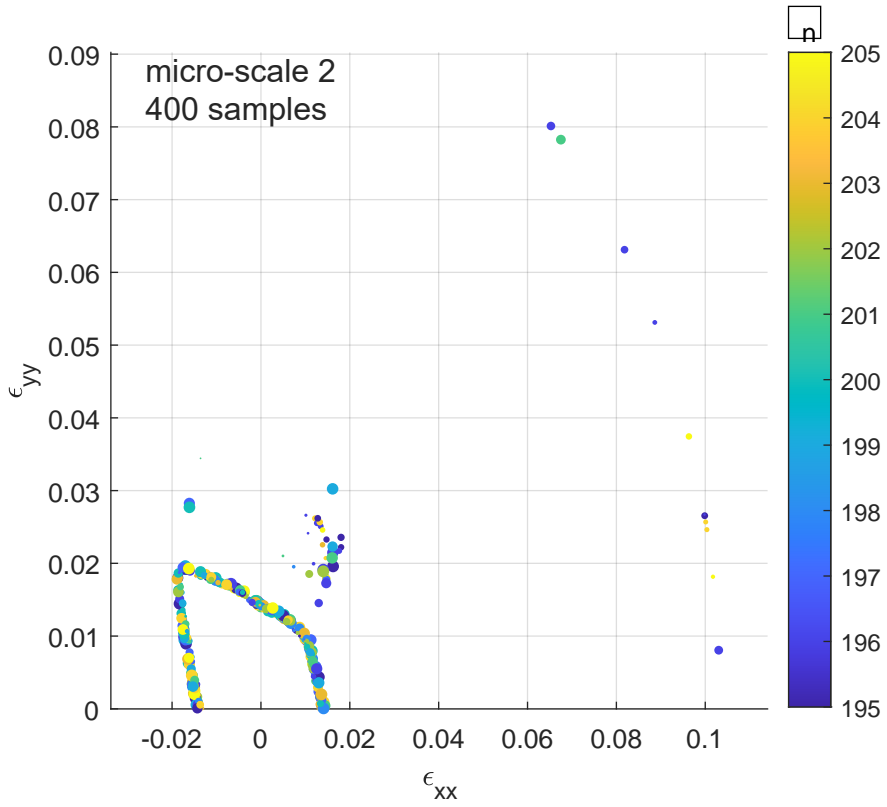

Supplement: Supplementary file 1 [file materials-15-00965-s001.zip › Figure S1b.pdf]

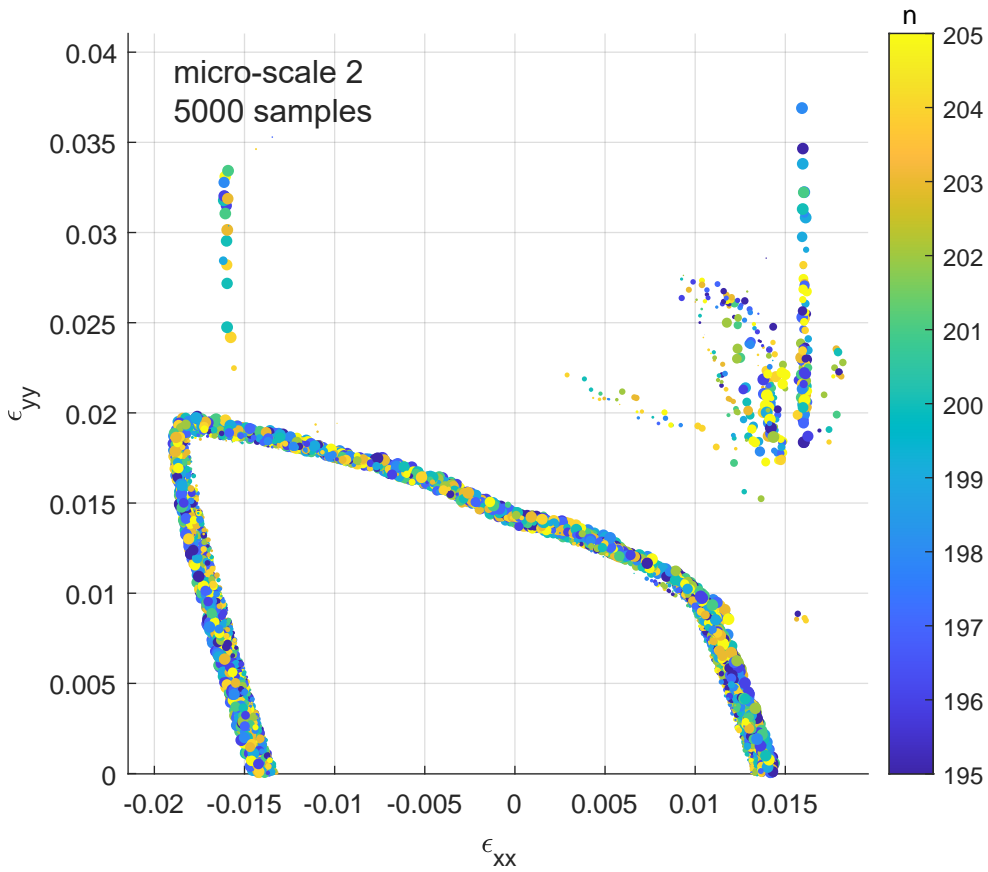

Supplement: Supplementary file 1 [file materials-15-00965-s001.zip › Figure S1c.pdf]

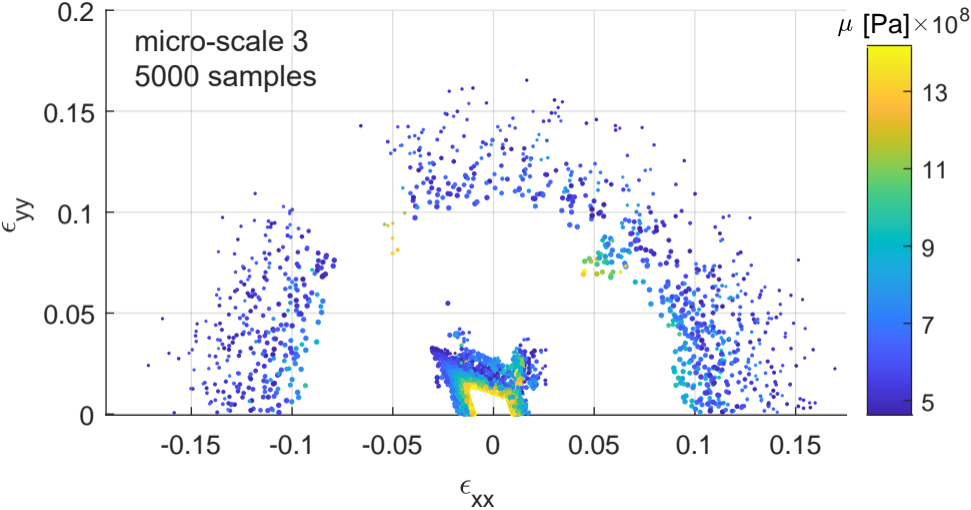

Supplement: Supplementary file 1 [file materials-15-00965-s001.zip › Figure S2.pdf]

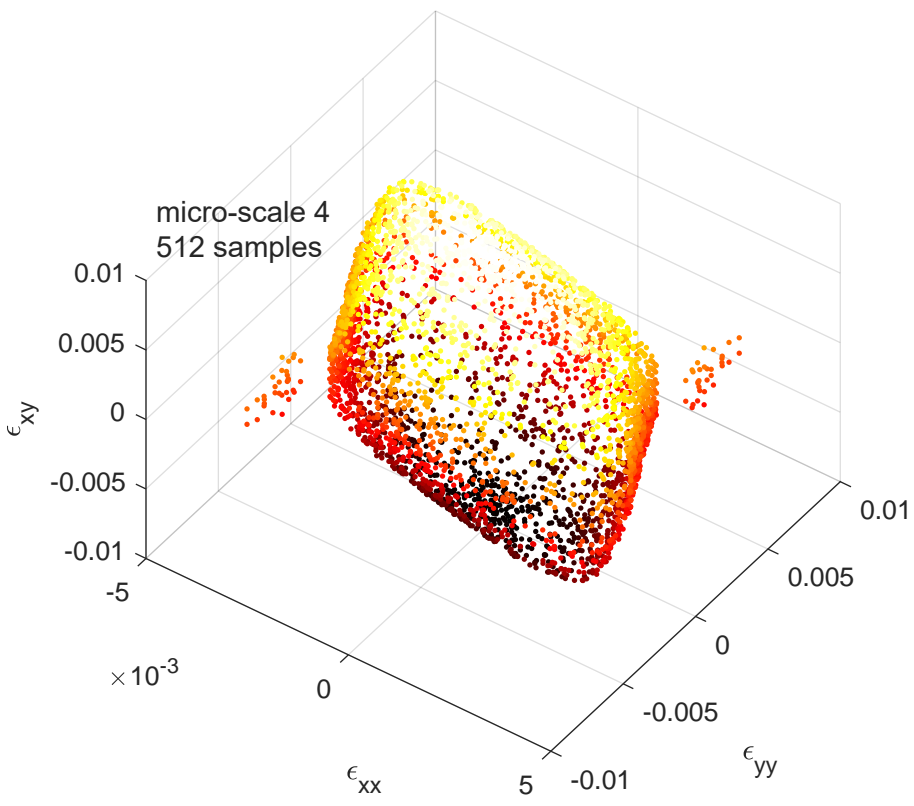

Supplement: Supplementary file 1 [file materials-15-00965-s001.zip › Figure S3a.pdf]

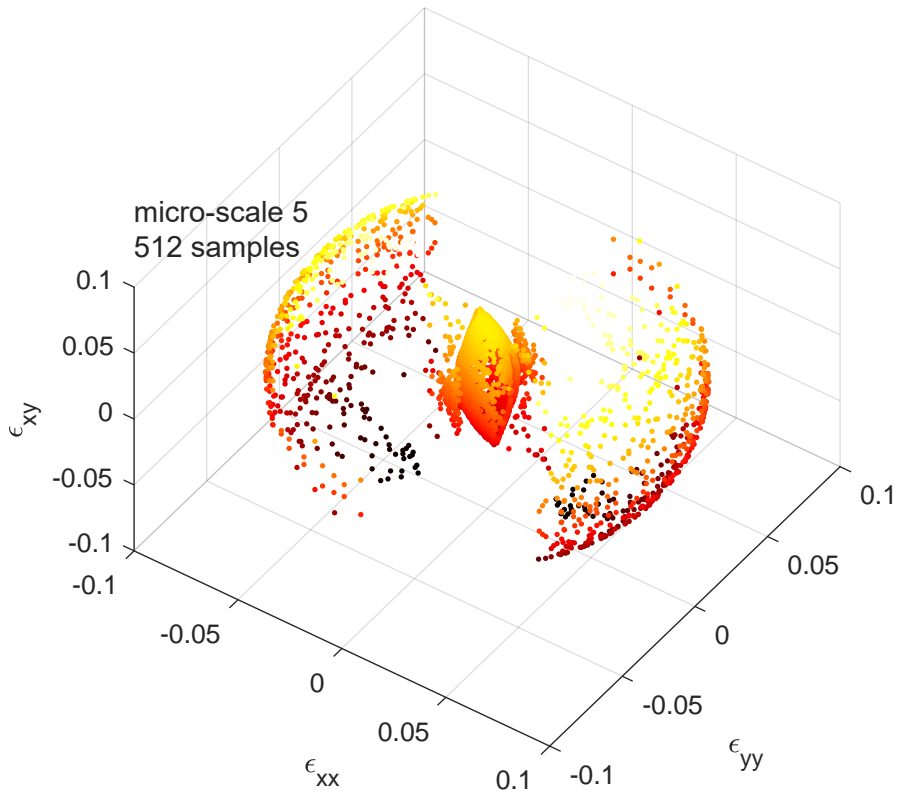

Supplement: Supplementary file 1 [file materials-15-00965-s001.zip › Figure S3b.pdf]

Regression model: Exponential GPR

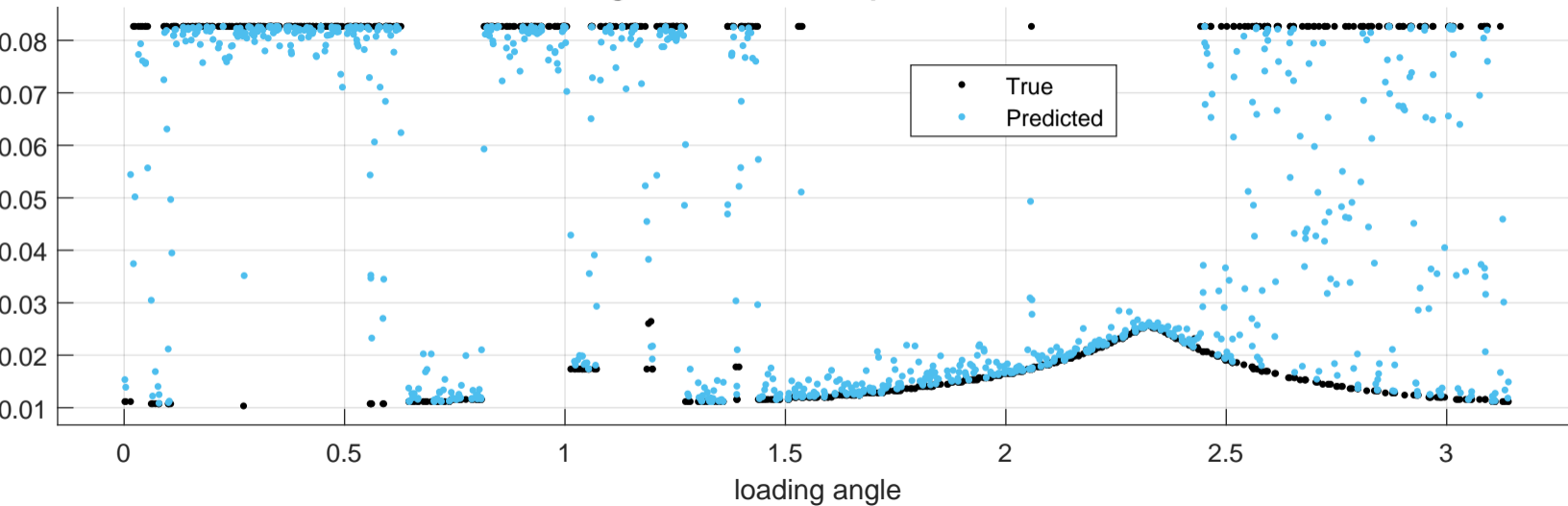

Supplement: Supplementary file 1 [file materials-15-00965-s001.zip › Figure S4.pdf]

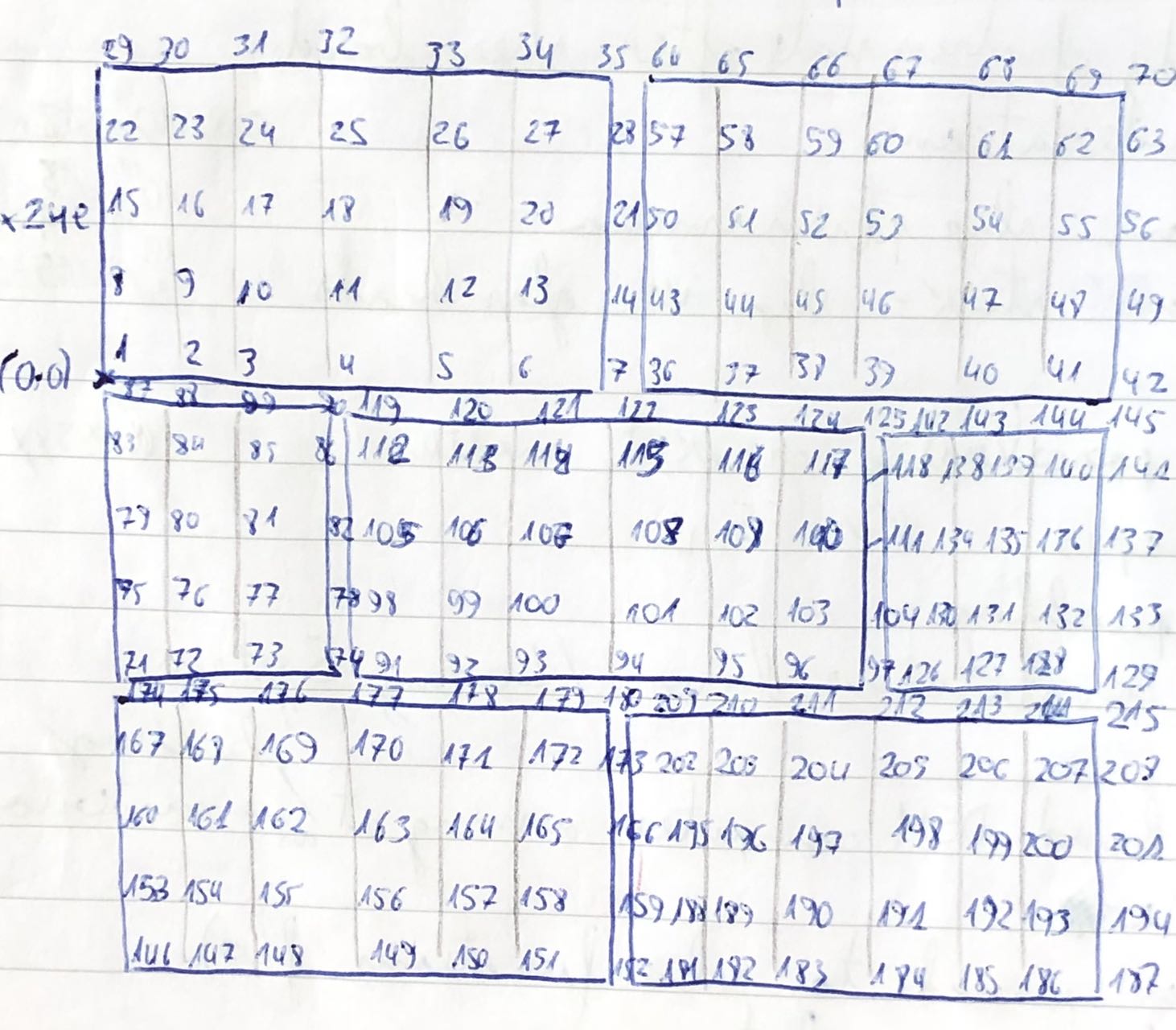

Supplement: Supplementary file 1 [file materials-15-00965-s001.zip › Node_numbering.jpg]
